# Draft Genome Sequence of Oil-Degrading Bacterium *Gallaecimonas pentaromativorans* Strain YA_1 from the Southwest Indian Ocean

**DOI:** 10.1128/genomeA.00764-16

**Published:** 2016-08-04

**Authors:** Yiyuan Xu, Chong Ren, Ruixuan Chen, Runying Zeng

**Affiliations:** a State Key Laboratory Breeding Base of Marine Biogenetic Resource, Third Institute of Oceanography SOA, Xiamen, People's Republic of China; b China Shipbuilding Industry Corporation (CSIC) No. 710 Institute, Wuhan, People's Republic of China; c Fujian Collaborative Innovation Center for Exploitation and Utilization of Marine Biological Resources, Xiamen, People's Republic of China

## Abstract

*Gallaecimonas pentaromativorans* has been previously reported to be capable of degrading crude oil and diesel oil. *G. pentaromativorans* strain YA_1 was isolated from the southwest Indian Ocean and can degrade crude oil. This study reports the draft genome sequence of *G. pentaromativorans*, which can provide insights into the mechanisms of microbial oil biodegradation.

## GENOME ANNOUNCEMENT

As reported previously, the genus *Gallaecimonas* is composed of only two species, *Gallaecimonas pentaromativorans* and *Gallaecimonas xiamenensis* (1). *G. pentaromativorans* was previously isolated from the Corcubión Ria in northwestern Spain, which was a coastal system that was heavily polluted by the Prestige oil spill in 2002 (2). It has been reported that *G. pentaromativorans* can grow vigorously with crude oil and slightly with diesel oil (3). Also, it was revealed that *G. pentaromativorans* can produce biosurfactants through experiments using the hemolytic test, drop collapse, and oil-spreading test, which can promote the oil degradation process by dispersing oil molecules (4). These research results demonstrated that *G. pentaromativorans* has the potential to treat oil pollution. The shotgun sequencing of the *Gallaecimonas pentaromativorans* genome sequence will facilitate the application of *Gallaecimonas* strains in the treatment of oil pollution.

*Gallaecimonas pentaromativorans* strain YA_1 was isolated from the deep sea of the southwest Indian Ocean at the depth of 2,168 m (49°44′E, 37°37′S). Strain YA_1 has been deposited in Marine Culture Collection of China (accession no. MCCC A11635). The genomic DNA of strain YA_1 was extracted using the bacterial genomic DNA extraction kit (BioTeke, Beijing, China), according to the instructions. The whole-genome shotgun project was performed on an Illumina MiSeq 2000 platform using paired-end (300 bp to ~500 bp) sequencing. First, the raw data were filtered to remove the unqualified data and then assembled using SOAP*denovo* version 2.04 (5). GapCloser version 1.12 (6) was used for local gap closure and base correction. The prediction of rRNA and tRNA was performed by Barrnap 0.4.2 (7) and tRNAscan-SE version 1.3.1 (8), respectively. Glimmer 3.02 (9) was used to predict the genes. The genes were annotated by a BLAST search against several databases, including NCBI-NR (nonredundant), string (version 9.05), Gene Ontology (GO), and Kyoto Encyclopedia of Genes and Genomes (KEGG) (10).

The draft genome of *G. pentaromativorans* strain YA_1 is composed of 86 scaffolds, with a total length of 4,325,653 bp and a G+C content of 58.25%; the largest contig length is 617,526 bp, and the contig *N*_50_ length is 206,153 bp. According to the Glimmer server, 3,917 genes of 3,821,355-bp total length were predicted. The average gene length is 975 bp, and the G+C content in the gene region is 59.5%. In total, there are 14 predicted enzymes that may be involved in the pathways related to oil degradation, including one *cis*-muconate cycloisomerase (EC 5.5.1.2), three acetyl-coenzyme A (CoA) acyltransferases (EC 2.3.1.16), one 3-hydroxybutyryl-CoA dehydrogenase (EC 1.1.1.157), two acetyl-CoA *C*-acetyltransferases (EC 2.3.1.9), two alcohol dehydrogenases (EC 1.1.1.1), one amidase (EC 3.5.1.4), one homogentisate 1,2-dioxygenase (EC 1.13.11.5), one maleylacetoacetate isomerase (EC 5.2.1.2), one fumarylacetoacetate (FAA) hydrolase (EC 3.7.1.2), and one glutaconate CoA-transferase (EC 2.8.3.12). Clusters of Orthologous Groups of proteins (COG) function classification revealed that there are 156 genes with unknown functions. They account for >9% of 1,724 sequences in total, which means *G. pentaromativorans* strain YA_1 deserves more exploration.

### Nucleotide sequence accession numbers.

This whole-genome shotgun project has been deposited at DDBJ/EMBL/GenBank under the accession no. LFWC00000000. The version described in this paper is version LFWC01000000.
